# Metabolic Analysis of the Development of the Plant-Parasitic Cyst Nematodes *Heterodera schachtii* and *Heterodera trifolii* by Capillary Electrophoresis Time-of-Flight Mass Spectrometry

**DOI:** 10.3390/ijms221910488

**Published:** 2021-09-28

**Authors:** Awraris Derbie Assefa, Seong-Hoon Kim, Vimalraj Mani, Hyoung-Rai Ko, Bum-Soo Hahn

**Affiliations:** 1 National Agrobiodiversity Center, National Institute of Agricultural Sciences, Rural Development Administration, Jeonju 54874, Korea; awraris@korea.kr (A.D.A.); shkim0819@korea.kr (S.-H.K.); 2 Department of Agricultural Biotechnology, National Institute of Agricultural Sciences, Rural Development Administration, Jeonju 54874, Korea; vimalraj08@gmail.com; 3 Crop Protection Division, National Institute of Agricultural Sciences, Rural Development Administration, Wanju 55365, Korea; reachsg@korea.kr

**Keywords:** cyst nematodes, *Heterodera schachtii*, *Heterodera trifolii*, metabolic pathways, CE-TOF/MS

## Abstract

The cyst nematodes *Heterodera schachtii* and *Heterodera trifolii*, whose major hosts are sugar beet and clover, respectively, damage a broad range of plants, resulting in significant economic losses. Nematodes synthesize metabolites for organismal development and social communication. We performed metabolic profiling of *H. schachtii* and *H. trifolii* in the egg, juvenile 2 (J2), and female stages. In all, 392 peaks were analyzed by capillary electrophoresis time-of-flight mass spectrometry, which revealed a lot of similarities among metabolomes. Aromatic amino acid metabolism, carbohydrate metabolism, choline metabolism, methionine salvage pathway, glutamate metabolism, urea cycle, glycolysis, gluconeogenesis, coenzyme metabolism, purine metabolism, pyrimidine metabolism, and tricarboxylic acid (TCA) cycle for energy conversion (β-oxidation and branched-chain amino acid metabolism) energy storage were involved in all stages studied. The egg and female stages synthesized higher levels of metabolites compared to the J2 stage. The key metabolites detected were glycerol, guanosine, hydroxyproline, citric acid, phosphorylcholine, and the essential amino acids Phe, Leu, Ser, and Val. Metabolites, such as hydroxyproline, acetylcholine, serotonin, glutathione, and glutathione disulfide, which are associated with growth and reproduction, mobility, and neurotransmission, predominated in the J2 stage. Other metabolites, such as SAM, 3PSer, 3-ureidopropionic acid, CTP, UDP, UTP, 3-hydroxy-3-methylglutaric acid, 2-amino-2-(hydroxymethyl-1,3-propanediol, 2-hydroxy-4-methylvaleric acid, Gly Asp, glucuronic acid-3 + galacturonic acid-3 Ser-Glu, citrulline, and γ-Glu-Asn, were highly detected in the egg stage. Meanwhile, nicotinamide, 3-PG, F6P, Cys, ADP-Ribose, Ru5P, S7P, IMP, DAP, diethanolamine, *p*-Hydroxybenzoic acid, and γ-Glu-Arg_divalent were unique to the J2 stage. Formiminoglutamic acid, nicotinaminde riboside + XC0089, putrescine, thiamine 2,3-dihydroxybenzoic acid, 3-methyladenine, caffeic acid, ferulic acid, *m*-hydrobenzoic acid, *o*- and *p*-coumaric acid, and shikimic acid were specific to the female stage. Overall, highly similar identities and quantities of metabolites between the corresponding stages of the two species of nematode were observed. Our results will be a valuable resource for further studies of physiological changes related to the development of nematodes and nematode–plant interactions.

## 1. Introduction

Nematodes, arguably one of the largest groups of animals, cause significant agricultural losses and human diseases [1,2]. *Heterodera schachtii* is considered a major pest in sugar beet production. It parasitizes more than 200 plant species, most notably the Chenopodiaceae and Cruciferae families [3]. It is a serious pathogen of Chinese cabbage in the highland fields of Korea [4,5]. *Heterodera trifolii* is a dark brown, large-cyst nematode with a cone top structure and a strong under-bridge with a sheaf-like organ in the center and heavily furcated ends within the fenestral area. It has a wide host range, including *Trifolium*, *Medicago*, *Lespedeza*, *Glycine*, *Dianthus*, *Lotus*, *Vinca*, *Melilotus*, *Spinacea*, *Polygonum*, *Rumex*, *Chenopodium*, *Agrostis pai*, *Curcuma larga*, *Solanum*, *Pisum*, and *Phaseolus* [6]. *H. schachtii* and *H. trifolii* have morphological resemblances, host range similarityies and exhibit similar symptoms of damage; this makes it difficult to distinguish between these species in the field [7,8].

Plant nematodes are soil-borne pathogens that mainly infect plant roots through sophisticated mechanisms of parasitism to manipulate the host, such as different mechanical stylets and secretion of effectors [9,10]. Effectors can be proteins and other varieties of non-protein metabolites. Some effectors, including coronatine (*Pseudomonas syringae*), phenylacetic acid (*Rhizoctonia solani*), spermine (*Heterodera schachtii*), sphingolipids (*Magnaporthe oryzae*), polysaccharides, putrescine (*Ralstonia solanacearum*), and toxA (*Pyreniphora tritici-repentis*) are summarized elsewhere [11]. Nematode-related plant diseases can be categorized as cyst nematodes (associated with the genera *Heterodera* and *Globodera*), root-knots (associated with the genus *Meloidogyne*), and lesions (associated with the genus *Pratylenchus*). *Heterodera schachtii* induces the formation of multinucleated cells, resulting in cellular imbalance in its host [12,13]. Infection by *Heterodera schachtii* influences stress-related hormones such as ethylene, jasmonic acid, abscisic acid, gibberellin, and amino acids in wheat and Arabidopsis [14,15,16,17,18].

Nematodes have fewer metabolites than plants. Some species, such as *Caenorhabditis elegans* and *Pristionchus pacificus*, produce nematode-derived modular metabolites (NDMMs) to regulate organismal development, lifespan, and social communication [19]. Several phospholipids are abundant in the filarial nematodes *Onchocerca volvulus*, *Onchocerca ochengi*, and *Litomosoides sigmodontis* [20]. The metabolic potential of nematodes is critical for their development, growth, and pathogenicity. Characterizing nematode biochemicals could provide insight into central aspects of nematode–plant interactions. However, discriminating between pathogen and plant metabolites in studies of nematode–plant interactions is challenging. In one study, the metabolomes of plant microbes were labeled with heavy isotopes using a targeted approach of metabolite detection [21]. In another study, the metabolic profiles of separated host and pathogens were analyzed and metabolic differences were detected [22]. However, this approach did not distinguish individual compounds. Specific metabolic databases for plant pathogens are lacking. Many research institutions possess an in-house chemical library specific to their research interest, which is not publicly available. The use of metabolomics to identify pathogenic compounds enhances the understanding of the nematode side of the plant–pathogen relationship. Profiling the metabolic potential of understudied species of nematode, such as *Heterodera schachtii* and *Heterodera trifolii*, can provide insight into their biology and molecular adaptations. Hence, we performed metabolic profiling of these two economically important plant-parasitic cyst nematode species in the egg, juvenile 2 (J2), and female stages. A stage-wise comparison of the metabolites in these two nematodes will facilitate studies of physiological changes related to the development of nematodes and nematode–plant interactions.

## 2. Results

### 2.1. Data Collection

According to capillary electrophoresis time-of-flight mass spectrometer (CE-TOF/MS), the metabolomes of the two nematodes (*H. schachtii* and *H. trifolii*) showed 392 detectable peaks (225 in cationic and 167 in anionic modes). The peaks were annotated based on the Human Metabolome Technology (HMT) (Yamagata, Japan) standard library and Known-Unknown peak library based on *m*/*z* values and migration time (MT). Detailed information of each peak is presented in Table S2. Selected data based on standardized relative area and the involvements of metabolites in major pathways are provided in other tables and figures. In all stages under investigation, almost equal numbers of detectable peaks were found in both species, with a slightly higher number in *H. schachtii* (380). The largest number of peaks was in the female stage (345 in *H. schachtii* and 357 in *H. trifolii*) followed by the egg stage (309 and 294 detectable peaks in *H. schachtii* and *H. trifolii*, respectively). The J2 stage had the fewest detectable peaks (295 and 286 in *H. schachtii* and *H. trifolii*, respectively).

### 2.2. Major Metabolic Biosynthetic Pathways in Nematode Species

**Figure 1:**
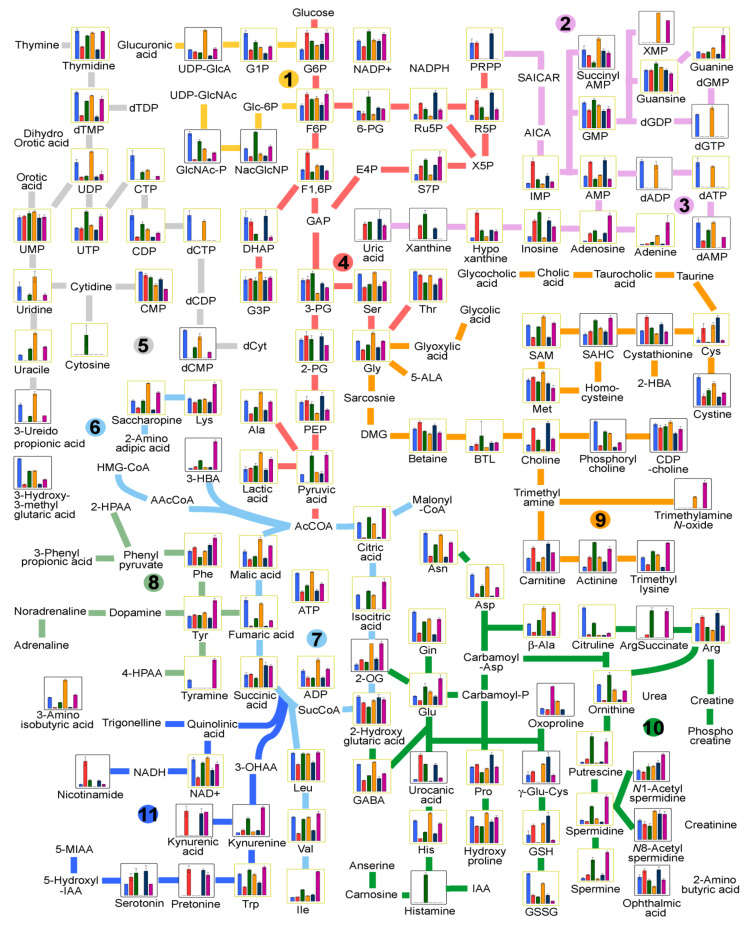
Integrated superimposed metabolic pathway map. Carbohydrate metabolism (**1**); purine metabolism (**2**,**3**); glycolysis and gluconeogenesis metabolism (**4**); pyrimidine metabolism (**5**); TCA cycle to store energy (**6**); TCA cycle for energy conversion: β-oxidation and BCAA metabolism (**7**); aromatic amino acid metabolism-phenylalanine and tyrosine (**8**); choline metabolism and methionine salvage (**9**); glutamate metabolism and urea cycle (**10**); and aromatic amino acid metabolism- tryptophan (**11**). The bars/lines represent peak areas of metabolites in *H. schachtii* egg (blue), *H. schachtii* juvenile 2 (red), *H. schachtii* female (green), *H. trifolii* egg (orange), *H. trifolii* juvenile 2 (mazarine), *H. trifolii* female (purple), respectively. Boxes with no bars indicate metabolites not detected. Refer Table S2 for the abbreviations.

The metabolites and intermediates were related to active energy metabolism, metabolites useful for growth and motility, cellular homeostasis, cell synthesis, amino acids, nucleic acids, and other properties (Table S1 and Figure 1). The metabolic biosynthetic pathways included aromatic amino acid metabolism, carbohydrate metabolism, choline metabolism, methionine salvage pathway, glutamate metabolism, urea cycle, glycolysis, gluconeogenesis, coenzyme metabolism, purine metabolism, pyrimidine metabolism, and tricarboxylic acid (TCA) cycle for energy conversion (β-oxidation and BCAA metabolism) and energy storage. Of the 392 peaks, the identity of 15 was unknown (Table S2). Among the 377 peaks explored at all stages of the two nematode species, 189 of the metabolites were involved in biosynthetic pathways (Table S2 and Figure 1). The remaining 188 metabolites were not involved in metabolic pathways (Table S2 and Figure S1).

### 2.3. Relative Peak Areas of Metabolites and Patterns of Variation across the Stages

All metabolic pathways were found at all stages of both nematode species. However, the levels of the metabolites varied across the stages. The largest number of metabolites (39) was detected in glutamate metabolism and urea cycle followed by purine metabolism (24) and TCA cycle (22). The numbers of metabolites related to choline metabolism, pyrimidine metabolism, aromatic amino acid metabolism, carbohydrate metabolism, metabolism of coenzyme, and glycolysis and gluconeogenesis metabolisms were 19, 19, 18, 16, 14, and 15, respectively. As shown in Table S1, some of the metabolites were related to more than one metabolic pathway. The relative areas of metabolites involved in the biosynthetic pathways are presented in Table S2 and also shown in Figure 1. Significantly higher levels (2- to 2.5-fold higher) of the sum of the relative areas of the metabolites in aromatic amino acid metabolism were observed in the female stage of *H. schachtii* compared to the other stages in both species. By contrast, the levels of the sum of the relative areas in carbohydrate metabolism, coenzyme metabolism, pyrimidine metabolism, and TCA cycle were 1.2- to 1.5-, 1.4- to 2.7-, 1.4- to 1.9-, and 2.8- to 3.6-fold lower, respectively, in the J2 stage than in the egg and female stages. Approximately 50% of the metabolites detected were not involved in the metabolic biosynthetic pathways. The peak areas of the metabolites not involved in biosynthetic pathways are presented in Figure S1.

Based on their levels at the developmental stages, the metabolites showed distinct patterns in both nematode species. In the first category, a relatively large number of metabolites were detected in egg and female stages compared to the J2 stage (highlighted orange in Table S2). The second category contained metabolites that were highly detected in egg stage (highlighted light blue in Table S2). The third category that contained a relatively smaller number of metabolites compared with other stages included those metabolites which were highly detected in J2 stage (highlighted light green in Table S2) while the fourth had metabolites highly detected in female stage (highlighted yellow in Table S2). On the other hand, several metabolites were detected exclusively in either of the species and in a single stage. For example, 1-methyluric acid, 2,4-diaminobutyric acid, 4-methyl-5-thiazoleethanol, 3-(4-hydroxyphenyl)propionic acid +2-(4-hydroxyphenyl)propionic acid + 3-(3-hydroxyphenyl)propionic acid, caffeic acid, ferulic acid, γ-Glu-Ile-2 + γ-Glu-Leu-2, *o*-coumaric acid + *p*-coumaric acid, and XA0012 were only detected in the egg stage of *H. trifolii*. The female stage of *H. schachtii* uniquely showed cytosine, histamine, 3-aminopropane-1,2-diol, 2-oxohexanoic acid + 2-oxoleucine + 2K3MVA, citramalic acid, and neopterin. 2,’3′-cCMP + cCMP and *N*-methylglutamic acid were detected only in the J2 stage of *H. schachtii*; whereas 4-amino-3-hydroybutyric acid and 2-amino-2-(hydroxymethyl)-1,3-propanediol were unique to the J2 stage of *H. trifolii* and the egg stage of *H. schachtii*, respectively.

All 20 essential amino acids were detected. Leu, Val, Ser, Phe, Ile, Gly, Thr, and Met predominated based on the average values of relative peak areas in both species (Table S2). A similar trend was observed among the stages for the two species. Overall, the levels of most amino acids (Lys, Ala, Leu, Val, Trp, His, Glu, Gln, Asn, Asp, Gly, and Ser) were lower in the J2 stage than in other stages (Figure 1 and Table S2). The major metabolite in aromatic amino acid (AAA) metabolism was Phe (0.026 to 0.073 mg^−1^) followed by Tyr (0.0058 to 0.02 mg^−1^) in both species. The major metabolite in carbohydrate metabolism was the G3P (0.0035 to 0.0049 mg^−1^), which was proportionally distributed in all stages, followed by G6P (0.00064 to 0.0017 mg^−1^), pyruvic acid (0.00 to 0.0021 mg^−1^), and GlcNAc-P (0.00095 to 0.0018 mg^−1^). Phosphorylcholine (0.010 to 0.086 mg^−1^), Met (0.013 to 0.028 mg^−1^), glycerophosphocholine (0.011 to 0.023 mg^−1^), and cystathionine (0.011 to 0.029 mg^−1^) were the major metabolites of choline in descending order. Hydroxyproline (0.032 to 0.085 mg^−1^), guanosine (0.047 to 0.074 mg^−1^), and uridine monophosphate (UMP) (0.0019 to 0.0025 mg^−1^) predominated in glutamate metabolism and the urea cycle, purine metabolism, and pyrimidine metabolism, respectively. The glycolysis and gluconeogenesis metabolic pathways were dominated by Ser (0.028 to 0.059 mg^−1^), Gly (0.019 to 0.049 mg^−1^), Thr (0.020 to 0.036 mg^−1^), lactic acid (0.018 to 0.045 mg^−1^), and malic acid (0.0054 to 0.021 mg^−1^). Several metabolites in vitamins metabolism (B1, B2, B3, B5, B6, B9 and C) were detected, compared to no vitamin B7 metabolite. Overall, threonic acid (0.00015 to 0.0013 mg^−1^), panthothenic acid (0.00042 to 0.00092 mg^−1^), and ascorbic acid (0.00 to 0.0015 mg^−1^) were the dominant metabolites of the coenzyme metabolic biosynthetic pathway. Leu (0.036 to 0.062 mg^−1^), Val (0.016 to 0.060 mg^−1^), Ile (0.013 to 0.046 mg^−1^), and citric acid (0.0017 to 0.059 mg^−1^) were synthesized in higher levels in the TCA cycle. Among the metabolites not included in any of the metabolic pathways described above, glycerol (0.048 to 0.077 mg^−1^) and lactic acid (0.019 to 0.045 mg^−1^) predominated.

### 2.4. Highly Detected Metabolites in Nematode Species

**Figure 2:**
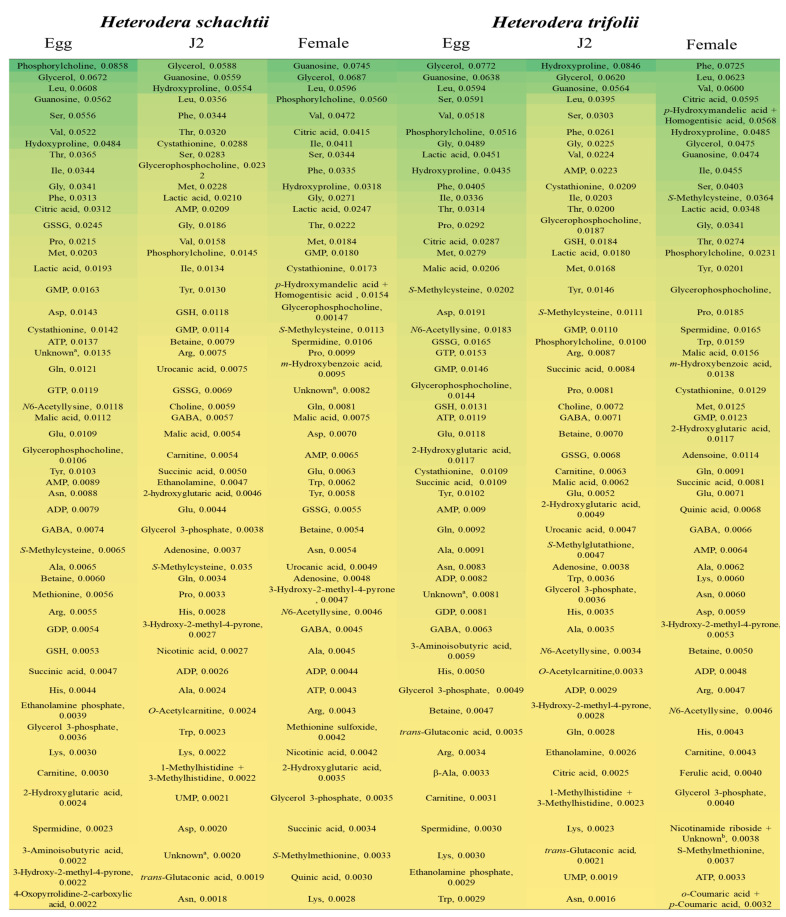
Top fifty metabolites in the egg, juvenile 2 (J2), and female stages of *H. schachtii* and *H. trifolii* nematode species arranged in descending order (top to bottom) of relative peak area (mg^−1^). Refer to Table S2 for abbreviations of metabolites. The unknown metabolites XA0033 (^a^) (S/NO, 355; Table S2) and XC0089 (^b^) (S/No, 365; Table S2) had predicted *m*/*z* values of 242.080 and 255.099, respectively.

The levels of the metabolites according to stage are listed in Table S2. The top 50 metabolites based on the relative area at each stage are shown in Figure 2. Phosphorylcholine, glycerol, Leu, guanosine, Ser, and Val were among the highly detected metabolites in the egg stage of *H. trifolii* and *H. schachtii*, although their levels differed between the two species. In the J2 stage, the highly detected metabolites included glycerol, guanosine, hydroxyproline, Leu, Phe, and Ser. There was a slight difference in the levels of highly detected metabolites in the female stage between the two species. The top five highly detected metabolites in the female stage in *H. schachtii* were guanosine, glycerol, Leu, phosphorylcholine and Val, whereas Phe, Leu, Val, citric acid *p*-hydroxymandelic acid + homogentisic acid (in decreasing order) were the most detected for for *H. trifolii*. Overall (Figure 2), Leu, glycerol, and guanosine were highly detected metabolites at all stages, except in the female stage of *H. trifolii* (ninth and tenth, respectively).

### 2.5. Pearson’s Correlation and Multivariate Analysis of Metabolic Profiles

**Figure 3:**
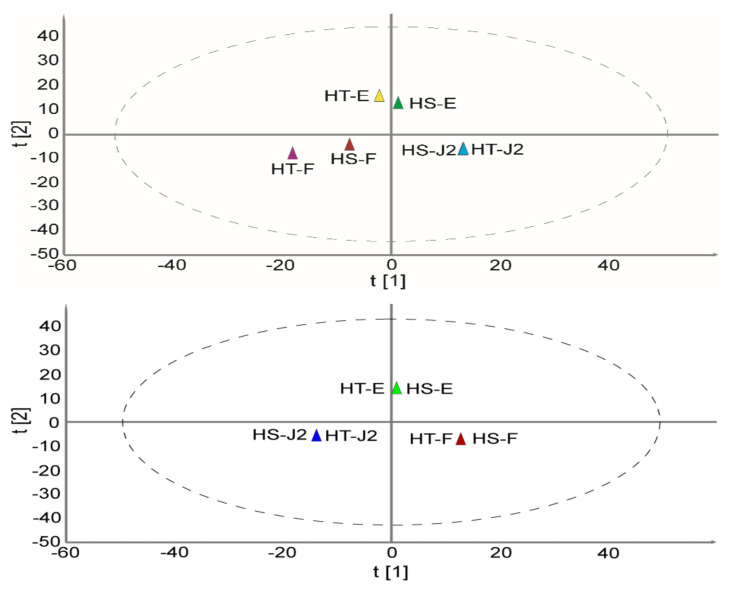
Score plots of the principal component analysis (PCA) (**top**) and orthogonal partial least squares discriminant analysis (OPLS-DA) (**bottom**) of *H. schachtii* and *H. trifolii* based on the relative peak areas of metabolites. HS-E, *H. schachtii* egg stage; HS-J2, *H. schachtii* juvenile 2 stage; HS-F, *H. schachtii* female stage; HT-E, *H. trifolii* egg stage; HT-J2, *H. trifolii* juvenile 2 stage; and HT-F, *H. trifolii* female stage.

To study the relationship between the samples based on their metabolic profile, Pearson’s correlation analysis (Table 1) was performed using SPSS at *p* < 0.01. A correlation coefficient of 0.7481 to 0.9651 was obtained, indicating meaningful associations between metabolites among the samples. Stage-wise correlation was highly significant for HS-J2 vs. HT-J2 (r = 0.9651) and HS-egg vs. HT-egg (r = 0.9545). The data in Table S2 were subjected to an unsupervised principal component analysis (PCA). The first three components (PC1, PC2, and PC3), which had eigenvalues of 2.27, 1.75, and 1.08, respectively, explained 85.1% (37.8, 29.2 and 18.1%, respectively) of the total variation (Figure 3). PCA and orthogonal partial least squares discriminant analysis (OPLS-DA) were performed to investigate similarities and/or differences between samples. The samples were associated based on their developmental stage. Loading scatter plots of PCA and OPLS-DA representing relative peak areas of the metabolites are presented in Figures S2 and S3. The absolute values of the loadings indicate the degree of contribution to the principal components. AMP (S/No 80 in Table S2), PRPP (S/No 285), betaine (S/No 92), and acetylcholine (S/No 67) were the greatest contributors to PC1 (positively) and the J2 stage, whereas trimethyllysine (S/No 246), citric acid (S/No 109), isocitric acid 9S/No 204), and homocitrulline (S/No 189) negatively contributed to PC1 and were highly associated with the female stage. The highest greatest contributors to PC2 were dGTP (S/No 134), GTP (S/No 180), 3-ureidoisobutyric acid (S/No 43), dADP (S/No 124), and GDP (157), indicating strong associations with the egg stage (Figure S2). The variable important in projection (VIP) scores represent the metabolites’ contributions to the discrimination analysis; larger values indicate greater contributions. The VIP values of the metabolites are presented in Table S3. More than 213 metabolites had VIP scores of >1. The purine metabolic biosynthetic pathway products, dADP, dATP, dGTP, GTP, ATP, ADP, and 2′deoxyguanosine made the greatest contribution to the discrimination analysis, with VIP values of 1.31033, 1.30812, 1.3009, 1.29586, 1.29409, 1.29038, and 1.28661, respectively. Deoxycytidine triphosphate (dCTP), which contains a pyrimidine base, was also among the greatest contributors (VIP 1.29811, ranking fourth).

The difference in the levels of metabolites between the stages of the two species was evaluated based on *p* values computed using Welch’s test on relative peak areas (Table 2). In the egg stage, the relative areas of 92 metabolites were not significantly different in the two species, but 68 were significantly higher in *H. schachtii* and 94 metabolites had relative areas significantly higher in *H. trifolii* than *H. schachtii*. In the egg stage, a large number of metabolites (132) showed no significant difference in relative peak areas between *H. trifolii* and *H. schachtii* in the J2 stage. This number was 122 in the female stage. The number of metabolites with significantly higher relative peak areas in *H. schachtii* compared to *H. trifolii* was 67 and 59 in the J2 and female stages, respectively. In addition, 59 peaks had relative areas significantly higher in the J2 stage of *H. schachtii* than *H. trifolii*. Unlike the egg and J2 stages, a large number of metabolites (128) showed significantly higher relative peak areas in the female stage of *H. trifolii* than *H. schachtii*.

### 2.6. Heat Map and Biological Properties of Stage-Specific Metabolites

**Figure 4:**
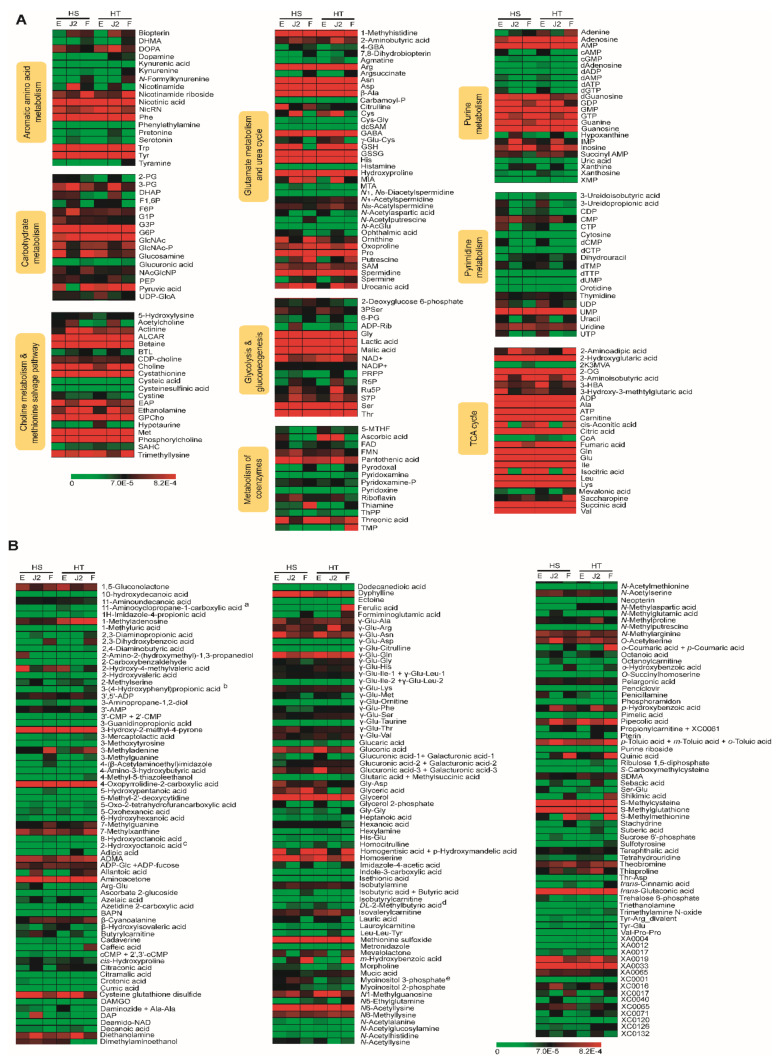
Heat map of changes in metabolite levels at the egg, J2, and female stages of *H. schachtii* (HS) and *H. trifolii* (HT). (**A**) metabolites involved in the metabolic pathways. (**B**) Metabolites not involved in the biosynthetic pathways. Comparative heat maps were constructed after quantile normalization of relative peak areas in the range of 0.0000 to 0.0.00082. Red indicates increased metabolite levels, green represents decreased levels, and black indicates intermediate levels. The heat map was generated using MeV (ver. 4.9.0) software. HS Egg, *H. schachtii* egg stage; HS J2, *H. schachtii* juvenile 2 stage; HS F, *H. schachtii* female stage; HT Egg, *H. trifolii* egg stage; HT J2, *H. trifolii* juvenile 2 stage; and HT female, *H. trifolii* female stage. Refer to Table S2 for abbreviations of metabolites.

The relative peak areas of the metabolites were quantile transformed and plotted on a heat map (Figure 4) with relative values of 0.00000 to 0.00082. Values are in red if the levels of metabolites were increased and in yellow if the levels were decreased. Black indicates an intermediate level. A large number of metabolites were detected at all the stages in both species and at comparable levels. As shown in Figure 4, more than 61 metabolites were highly detected at all stages. The metabolites highly detected at all stages of both species were nicotinic acid, Phe, Trp, and Tyr in aromatic amino acid metabolism; G3P and G6P in carbohydrate metabolism; ALCAR, betaine, cysthathionine, GPCho, Met, and phophorylcholine in choline metabolism and the methionine salvage pathway; Arg, Asn, Asp, β-Ala, GABA, GSSH, His, hydroxyproline, Pro, and spermidine in glutamate metabolism and the urea cycle; Gly, lactic acid, malic acid, Ser, and Thr in glycolysis and gluconeogenesis; adenosine, AMP, GMP, GMP, GTP, guanine, and guanosine from purine metabolism; UMP in pyrimidine metabolism; and ADP, Ala, ATP, carnitine, citric acid, fumaric acid, Gln, Glu, Ile, Leu, Lys, succinic acid, and Val from the TCA cycle. Overall, the TCA cycle metabolites predominated over other metabolic pathways. Other metabolites not included in the suggested pathway map (Figure 4b) and highly detected in both nematode species included 3-hydroxy-2-methyl-4-pyrone, 4-oxopyrrolidine-2-carboxylic acid, ADMA, aminoacetone, glycerol, homoserine, methionine sulfoxide, N6-acetyllysine, S-methylcysteine, S-methylglutathione, trans-glutaric acid, and XA0033 (*m/z* 242.080).

**Figure 5:**
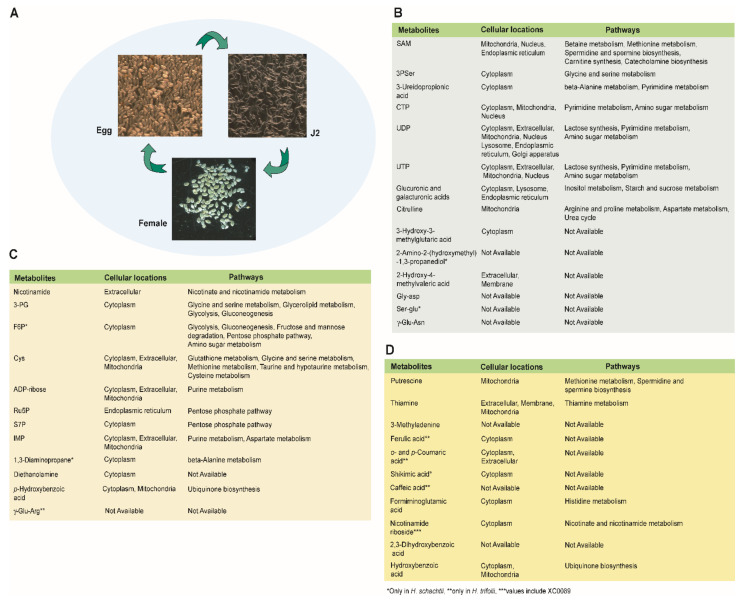
Stage-specific metabolites in *H. schachtii* and *H. trifolii* with their functions and biosynthetic pathways. Photographs of nematodes in the egg, J2, and female stages (**A**); metabolites highly detected at only the egg stage (**B**); J2 stage (**C**); and female stage (**D**). Cellular locations and pathways as defined in HMDB.

Some metabolites were dominant in a stage-specific manner. For example, ornithine, threonic acid, dTMP, 3-aminoisobutyric acid, *cis*-aconitic acid, homogentisic acid + *p*-hydroxymandelic acid, isocitric acid, mevalolactone, and *S*-methylmethionine were highly detected in the egg and female stages. By contrast, about 10% of the metabolites were specific to a single stage. Those stage-specific metabolites and their proposed cellular locations and pathways are presented in Figure 5. SAM, 3PSer, CTP, UDP, UTP, 3-hydroxy-3-methylglutaric acid, 2-amino-2-(hydroxymethyl-1,3-propanediol (only in *H. schachtii*), 3-ureidopropionic acid, 2-hydroxy-4-methylvaleric acid, Gly Asp, glucuronic acid-3 + galacturonic acid-3, Ser-Glu (only in *H. schachtii*), citrulline, and γ-Glu-Asn were specific to the egg stage, whereas nicotinamide, 3-PG, F6P (only *H. schachtii*), Cys, ADP-Rib, Ru5P, S7P, IMP, DAP (only *H. schachtii*), diethanolamine, *p*-hydrobenzoic acid, and γ-Glu-Arg (only *H. trifolii*) were upregulated in the J2 stage. Formiminoglutamic acid, nicotinamide riboside + XC0089, putrescine, thiamine, 2,3-dihydroxybenzoic acid, 3-methyladenine, *m*-hydrobenzoic acid, caffeic acid (HT), ferulic acid (HT), *o*-coumaric acid + *p*-coumaric acid (HT), and shikimic acid (HT) were highly detected in female stages where the last four metabolites were only detected in *H. trifoli*.

## 3. Discussion

Transcriptomics, proteomics, and metabolomics provide useful information on the functions of nematodes. As the genome represents the potential function of a cell, the metabolome represents the expressed function. Metabolomics, unlike transcriptomics, provides information about the biological functioning of nematodes and other organisms. The bioanalytical techniques used for metabolomics studies include, but are not limited to, LC-MS, GC-MS and NMR spectroscopy.

Metabolites, including intermediates and end products, are produced during the growth of nematodes to serve in fundamental physiological processes, such as growth and reproduction. They play a key role as energy carriers in the cell. Metabolites are used in the formation of indispensable macromolecules, such as amino acids, via anabolic metabolism, acetic acids, and citric acids by catabolic reactions, or are converted into other vital factors such as coenzymes. There are few metabolome-profiling studies of *H. schachtii* and *H. trifolii*. However, earlier studies on other nematode species, such as *M. incognita*, provide insight into the metabolomes of other species including *H. schachtii* and *H. trifolii*. Myers and Krusberg [23] reported that alanine, asparagine, aspartic acid, glutamic acid, glutamine, glycine, serine, and tryptophan were synthesized by *M. incognita*. In another study, 15 amino acids were detected in surface-sterilized second-stage larvae of *M. incognita* [24]. HPLC was used to measure the ADP, ATP, and AMP contents of eggs and juveniles of *H. schachtii* to calculate the adenylate energy charge and thereby extract information on cyst viability and the efficacy of nematocidal fumigants [25,26]. The intermediate profiles of the tyrosine, tryptophan, and purine pathways in *Caenorhabditis elegans* were analyzed by HPLC coupled to electrochemical detection to study the effects of lead exposure on nematodes [27]. The metabolites (ascarosides) that constitute chemical communication in the model nematode *C. elegans* were characterized by ESI-MS/MS and GC-MS analytical techniques [28].

To evaluate the metabolite profiles in the egg, J2, and female stages of *H. schachtii* and *H. trifolii*, we adopted an established CE-TOF/MS-based untargeted metabolome analysis approach [29,30]. We found high similarities in the identities and quantities of metabolome between *H. schachtii* and *H. trifolii*. According to OPLS-DA (Figure 3), there was an overlap of the corresponding stages of the two species indicating a strong similarity in the identities and levels of metabolites. In addition, metabolite levels varied according to the developmental stage within a species.

The predominant metabolites (Figure 2) in *H. schachtii* and *H. trifolii* included glycerol, guanosine, hydroxyproline, citric acid, phosphorylcholine, and the essential amino acids Phe, Leu, Ser. and Val. To adapt to extreme osmotic stress, nematodes accumulate or lose osmolytes. The high level of the organic osmolyte glycerol in *H. schachtii* and *H. trifolii* could be the result of cellular osmotic homeostasis, as in *Caenorhabditis elegans* [31]. Nematodes are capable of losing all intracellular or bound water during desiccation, using glycerol as replacement [32], leading to an increase in its level. The purine metabolite, guanosine, which protects against degenerative diseases [33] and has neuroprotective properties [34], reportedly modulates the glutamatergic system during basal conditions and excitotoxicity in *C*. *elegans* [35]. Hydroxyproline, a major protein in vertebrates, is important for oxidative folding and for the bioactivity of conotoxins [36]. The other highly detected choline metabolism product, phosphorylcholine, is a structural component of eukaryotes and prokaryotes and modulates host–pathogen interactions, such as invasion mechanisms and long-term persistence with low mortality [37,38,39,40]. Phosphorylcholine is also associated with polysaccharide components of the cell wall and cell membrane attached directly to sugar residues and generally called *N*-acetylgalactosamine [38], which was also detected in this study. The high amount of phosphorylcholine as well as the presence of glycans (glycosidically linked monosaccharides) in *H. schachtii* and *H. trifolii* suggests that these organisms could be used for biosynthetic and structural studies of polysaccharides [37,38]. Citric acid, which is involved in carbon fixation in prokaryotes, was among the highly detected metabolites in the egg and female stages. Citric acid reduced oxidative damage and protected against heavy metal stress in *C. elegans* [41].

The high amount of hydroxyproline, a component of collagen, the most abundant protein in animals [42], in the J2 stage (Figure 1 and Table S2) could be related to their motile nature that requires key muscle development. Other metabolites highly detected in the J2 stage included AMP and IMP, as reported in *M. incognita* [2]. Higher levels of glutathione (GSH) and its oxidized form (glutathione divalent, GSSG) were detected in the J2 and egg stages, respectively, compared to the female stage. Levels of GSH and GSSG were high in the egg stage, followed by the J2 stage. Baldacci-Cresp et al. [43] reported that GSH is essential for biotrophic interaction of nematodes with their host plants in the re-differentiation of root cells into multinucleate, hypertrophied giant cells (essential for nematode growth and reproduction). Hence, the increase in the levels of these metabolites in the egg and J2 stages might be triggered in preparation for the regulation of giant cell metabolism. Neuropeptides with neuromodulatory effects on muscular activity in nematode species [44,45], such as GABA, acetylcholine, dopamine, and serotonin, were detected in both *H. schachtii* and *H. trifolii*. The acetylcholine and serotonin levels were significantly higher in the J2 stage compared to the egg and female stages, which could be related to its motile nature. GABA and dopamine were reported in the J2 stage of *M. incognita* [46,47]. The TCA cycle products 2-hydoxyglutaric acid and 2-oxoglutaric acid reportedly extend the lifespan of *C. elegans* by inhibiting ATP synthase, which decreases mitochondrial respiration and mechanistic target of rapamycin (mTOR) signaling [48]. Moderate levels of 2-hydoxyglutaric acid and 2-oxoglutaric acid were detected at all stages of *H. schachtii*. The former was highly upregulated in the egg and female stages of *H. trifolii*. Another notable TCA cycle product, citric acid, was upregulated in the egg and female stages, and reportedly predominates in the egg stage of *M. incognita* [2]. Pyrimidine-related metabolites, such as 3-ureidopropionic acid, 3-ureidoisobutyric acid, CTP, cytosine, dCMP, UTP, Uracil, dUPM, dCTP and dTTP, were more abundant in the J2 stage of *H. schachtii* compared to *H. trifolii*.

Overall, strong similarities in metabolite identity and quantity were observed between corresponding stages in the two species of nematode. The egg and female stages accumulated more metabolites than the J2 stage. By contrast, some metabolites were stage specific (Figure 5) which could dictate a specific function to the respective stages. The metabolic profiling of nematodes helps elucidate their phenotypic state under the specified condition and their potential value. CE-TOF/MS allowed the identification of a large number of anionic and cationic metabolites in nematodes. A simultaneous analysis of metabolites and intermediates of various biosynthetic pathways makes the method suitable for comprehensive metabolome analysis. This study provides information that may enable the identification of metabolites of interest in the pathways responsible for the various physiological functions of nematodes.

## 4. Materials and Methods

### 4.1. Sample Preparation

Samples were prepared as described previously [2]. Nematodes (*Heterodera schachtii* and *Heterodera trifolii*) were maintained in roots of Chinese cabbage plants (*Brassica rapa* subsp. *pekinensis*) in a greenhouse at 25 °C. The stages of the nematodes were carefully monitored by manual inspection using a stereomicroscope and collected in the egg, J2, and female stages. Egg samples were collected by sucrose gradient centrifugation (35% sucrose, 1500 rpm, room temperature) after hypochlorite treatment (10% NaClO) of ruptured cysts for 5 min to remove contamination. The wash solution containing the eggs was passed through a 25 μm mesh, trapping the eggs. J2 samples were obtained by hatching eggs at 25 °C for 5 days in distilled-deionized autoclaved water and filtering using 5–7 KIMTECH ScienceWipers^®^ (Yuhan-Kimberly Professional, Seoul, Korea) on a Petri dish. To collect female-stage samples, infected roots were washed several times to remove potential contamination, chopped, ground, and filtered through a 75 μm mesh. Next, the females were directly handpicked from the ground roots using forceps under a stereomicroscope and washed with distilled-deionized autoclaved water. The J2 and female stage samples were not treated with hypochlorite because of nematode rupture and paralysis.

Samples of nematodes collected in the egg, juvenile, and female stages were snap-frozen using liquid nitrogen and stored at −80 °C until extraction. The samples were extracted as described previously [2]. Briefly, 50 mg sample was placed in a 2.0 mL cryotube containing Zirconia beads (5 mm Ø × 1, 3 mm Ø × 5) and 500 µL methanol was added. Cryotubes were fixed to a beads-shocker and homogenized at 4000 rpm, for 60 s (at 4 °C) twice. Next, 500 µL chloroform and 200 µL MilliQ water were added to each tube and vortexed for 30 s to remove phospholipids liberated from cell membranes that could be adsorbed on the capillary wall and reduce CE performance. The extracts were centrifuged at 2300× *g* for 5 min (at 4 °C) and the upper aqueous layer was carefully removed (~400 µL) and added to a pre-washed (using 250 µL MilliQ water) microcentrifugal filter and centrifuged at 9100× *g* until the solution was completely filtered (4 to 6 h). The filtrates containing metabolites were dried under vacuum. The dried samples were mixed with 50% acetonitrile in water (*v*/*v*) containing internal standards (10 μM) (Table 3) and homogenized (1500 rpm, 120 s × 2). The supernatant (400 μL) was passed through 5 kDa cut-off filter (ULTRAFREE-MC-PLHCC, Human Metabolome Technologies, Yamagata, Japan) to remove macromolecules. The filtrate was centrifugally concentrated and resuspended in 25 µL ultrapure water immediately before measurement. All reagents used for extraction of metabolites were obtained from common commercial sources. Water was purified with MilliQ purification system (Millipore, Bedford, MA, USA) 

### 4.2. CE-TOF/MS Analysis of Metabolites

The metabolites were measured in cation and anion modes by capillary electrophoresis-time-of-flight mass spectrometry (CE-TOF/MS) system (Agilent Technologies Inc., Santa Clara, CA, USA) [29,30,49]. Peaks were extracted using automatic integration software (MasterHands ver. 2.13.0.8.h; Keio University, Tokyo, Japan). The CE system was equipped with an Agilent 6210 TOF mass spectrometer, Agilent G1603A CE-MS Adapter Kit, Agilent 1100 Isocratic HPLC Pump, and Agilent G1607A CE-ESI-MS Sprayer Kit (Agilent Technologies, Waldbronn, Germany). The system was controlled by Agilent G2201AA ChemStation software version B.03.01 for CE (Agilent Technologies Inc., Santa Clara, CA, USA). A fused silica capillary column with 50 µm i.d × 80 cm total length (Agilent Technologies, Waldbronn, Germany) was used for separation. Analytical conditions were set as follows: run buffer (cation buffer solution (p/n: H3301–1001) and anion run buffer (p/n: I3302–1023), which were also used for rinsing; MS ionization was conducted in positive and negative ion modes; sample injection pressure was 50 mbar for 10 s; CE voltage was 27 kV (cation) and 30 kV (anion); MS capillary voltage was 4000 V (cation) and 3500 V (anion); and the MS scan range was *m*/*z* 50–1000.

### 4.3. Data Processing and Analysis

Peak information such as migration time (MT), peak area, and *m*/*z* value, were obtained using automatic integration software. Signal peaks corresponding to isotopomers, adduct ions, and other product ions of known metabolites were excluded. All signal peaks potentially corresponding to authentic compounds were extracted, and their migration time was normalized to those of the internal standards. Thereafter, the alignment of peaks was performed according to the *m*/*z* values and normalized MT values. Finally, relative peak areas were calculated against those of the internal standards, methionine sulfone (MetSul), and d-camphor-10-sulfonic acid (CSA) for cations and anions, respectively. The resultant relative area values were further calculated by sample amount. The peak area was converted into relative peak area using Equation (1). The peak detection limit was determined based on the signal-noise ratio; S/N = 3.

(1) $$\mathrm{Relative}\;\mathrm{Peak}\;\mathrm{Area}=\;\frac{\mathrm{Metabolite}\;\mathrm{Peak}\;\mathrm{Area}}{\mathrm{Internal}\;\mathrm{Standard}\;\mathrm{Peak}\;\mathrm{Area}\;\times \;\mathrm{Sample}\;\mathrm{Amount}\;}$$

Putative metabolites were assigned from the HMT standard library (Human Metabolome Technologies, Inc., Tsuruoka, Japan) and the Known-Unknown peak library based on *m*/*z* and MT values. The tolerance was ± 0.5 min for MT and ± 10 ppm for *m*/*z* (Equation (2)). If several peaks were assigned the same candidate, and the candidate was given the branch number.

(2) $$\mathrm{Mass}\;\mathrm{error}\;\left(\mathrm{ppm}\right)=\;\frac{\mathrm{Measured}\;\mathrm{Value}-\mathrm{Theoretical}\;\mathrm{Value}}{\mathrm{Measured}\;\mathrm{Value}}\;\times \;10^{6}$$

### 4.4. Statistical Analysis

We performed three biological replicates per sample. Values are averages of triplicates. Pearson’s correlation analysis and Welch’s test were performed using SPSS v. 25.0 software (SPSS Inc., Chicago, IL, USA). Principal component analysis (PCA) and OPLS-DA were performed in SIMCA v. 13.0.3 (Umetrics, Umea, Sweden). Quantitative values of metabolites were normalized using the normalization quantiles function in the R/Bioconductor-package preprocessCore [50] and heat maps were generated using MeV software v. 4.9.0 [51].

### 4.5. Plotting on the Pathway Map

The profiles of peaks with putative metabolites were represented on a metabolic pathway map using Visualization and Analysis of Networks containing Experimental Data (v. 5) software [52]. The pathway map was prepared based on the metabolic pathways of human cells.

## Figures and Tables

**Table 1:** Pearson’s correlation analysis of *H. schachtii* and *H. trifolii* in the egg, juvenile 2 (J2), and female (F) stages based on the relative peak areas of metabolites.

	HS-E	HS-J2	HS-F	HT-E	HT-J2	HT-F
HS-E	1					
HS-J2	0.8203 *	1				
HS-F	0.9417 *	0.8450 *	1			
HT-E	0.9545 *	0.8594 *	0.9357 *	1		
HT-J2	0.7975 *	0.9651 *	0.8103 *	0.8394 *	1	
HT-F	0.8008 *	0.7545 *	0.8814 *	0.8509 *	0.7481 *	1

* Correlation is significant at *p* < 0.01. HS-E, *H. schachtii* egg stage; HS-J2, *H. schachtii* juvenile 2 stage; HS-F, *H. schachtii* female stage; HT-E, *H. trifolii* egg stage; HT-J2, *H. trifolii* juvenile 2 stage; and HT-F, *H. trifolii* female stage.

**Table 2:** Comparison of the relative peak areas of metabolites between *H. schachtii* (HS) and *H. trifolii* (HT).

Stage	Metabolites with No Significant Difference between HS and HT	Metabolites Significantly Higher in HS than HT	Metabolites Significantly Higher in HT than HS
Egg	XC0120, Leu-Leu-Tyr, Lauric acid, Spermine, dCMP, Val-Pro-Pro, Gly-Asp, *N*-Methylproline, γ-Glu-Ile + γ-Glu-Leu, Glycerol 2-phosphate, Dimethylaminoethanol, dATP, Decanoic acid, *N*-Acetylhistidine, *N*-Acetylalanine, Pyridoxamine-P, 2-Methylserine, dADP, ATP, Dyphylline, Octanoic acid, dTMP, XC0071, Hexanoic acid, Pantothenic acid, *N*-Acetylaspartic acid, *N*-Acetylmethionine, Citric acid, XC0126, γ-Glu-Ornitine, *cis*-Hydroxyproline, 2-Aminoisobutyric acid + 2-Aminobutyric acid, Thymidine, Asn, Pelargonic acid, S7P, XA0065, Lys, γ-Glu-Gln, Leu, XA0017, γ-Glu-Ala, Ile, Isocitric acid, γ-Glu, Asn, Val, Gly-Gly, 8-Hydroxyoctanoic acid + 2-Hydroxyoctanoic acid, Tyr, Aminoacetone, 1-Methylhistidine + 3-Methylhistidine, dAMP, γ-Glu-Lys, β-Hydroxyisovaleric acid, NADP+, γ-Glu-Asp, ADP, Carnitine, Triethanolamine, Terephthalic acid, *p*-Toluic acid + *m*-Toluic acid+ *o*-Toluic acid, 4-Oxopyrrolidine-2-carboxylic acid, Ser, dGuanosine, NAD+, Glu, γ-Glu-Ser, CDP-choline, γ-Glu-Val, AMP, MTA, Tetrahydrouridine, *N*-Acetyllysine, *N*-Acetylserine, ADMA, ADP-Glc + GDP-fucose, Isobutylamine, Glycerol, Heptanoic acid, *O*-Succinylhomoserine, cAMP, Theobromine, 11-Aminoundecanoic acid, *N*-Acetylglucosylamine, 7-Methylguanine, FAD, Xanthosine, Azelaic acid, Diethanolamine, UTP, Glucaric acid, and 5-MTHF	Citrulline, Methionine sulfoxide, Urocanic acid, XC0132, Penicillamine, XC0065, MIA, Choline, 3-PG, Thiamine, Myoinositol 1-phosphate + Myoinositol 3-phosphate, Imidazole-4-acetic acid, CTP, PEP, Ethanolamine, Ophthalmic acid, CDP, GlcNAc-P, Myoinositol 2-phosphate, G1P, Ser-Glu, Acetylcholine, 5-Hydroxylysine, TMP, Putrescine, XC0016, Arg-Glu, Octanoylcarnitine, 3-Methyladenine, CMP, NAcGlcNP, 3-Hydroxy-3-methylglutaric acid, 3′,5′-ADP, 2-Deoxyglucose 6-phosphate, XA0033, Phosphorylcholine, 6-PG, Arg, 6-Hydroxyhexanoic acid, 2-Hydroxy-4-methylvaleric acid, Riboflavin, Ru5P, γ-Glu-Arg, XA0019, GSSG, Cysteine glutathione disulfide, 4-GBA, Ornithine, Inosine, EAP, Gln, Threonic acid, Cystathionine, Betaine, Cystine, 7-Methylxanthine, ThPP, His-Glu, γ-Glu-Thr, *N*-Acetylgalactosamine + ManNA + GlcNAc, IMP, XC0089 + Nicotinamide riboside, GABA, 3-Hydroxy-2-methyl-4-pyrone, Thr, GMP, Hydroxyproline, and FMN	Adenine, S-Methylmethionine, Cys, Hypotaurine, Uracil, 2-Hydroxyglutaric acid, *trans*-Glutaconic acid, b β-Cyanoalanine, Thiaproline, Quinic acid, Actinine, 1-Methyladenosine, Pipecolic acid, Glucuronic acid-1 + Galacturonic acid-1, Dihydrouracil, *S*-Methylcysteine, UDP-GlcA, *N*1-Methylguanosine, Glucuronic acid-2 + Galacturonic acid-2, 3-Aminoisobutyric acid, Isovalerylcarnitine, γ-Glu-Cys, GSH, 3-HBA, Oxoproline, β-Ala, Lactic acid, Succinic acid, Mevalonic acid, Adenosine, *S*-Methylglutathione, Malic acid, dcSAM, Mucic acid, *O*-Acetylhomoserine + 2-Aminoadipic acid, Fumaric acid, 3-Ureidopropionic acid, SDMA, Daminozide + Ala-Ala, 1,5-Gluconolactone, Succinyl AMP, γ-Glu-Citrulline, Glucuronic acid-3 + Galacturonic acid-3, Guanine, CoA, γ-Glu-His, 1-Aminocyclopropane-1-carboxylic acid +, Homoserinelactone, UDP, SAHC, Saccharopine, *N*6-Acetyllysine, Mevalolactone, 2-OG, DOPA, NicRN, F6P, GDP, *N*8-Acetylspermidine, *O*-Acetylserine, Gluconic acid, *N*1-Acetylspermidine, Gly, Homogentisic acid + *p*-Hydroxymandelic acid, F1,6P, ALCAR, *p*-Hydroxybenzoic acid, G6P, SAM, 3PSer, *N*6, Methyllysine, Ala, N^ω^-Methylarginine, Met, Trp, G3P, GPCho, Pro, γ-Glu-Phe, Asp, γ-Glu-Gly, Spermidine, Phe, 3′-AMP, GTP, dGTP, UMP, *cis*-Aconitic acid, Uridine, Nicotinic acid, Homoserine, Trimethyllysine, His, Guanosine, and γ-Glu-Met
J2	*p*-Toluic acid + *m*-Toluic acid + *o*-Toluic acid, Myoinositol 2-phosphate, XA0017, XC0071, Dihydrouracil, XC0065, ThPP, Xanthine, TMP, dTMP, GPCho, Gluconic acid, 3-PG, γ-Glu-Asn, Penciclovir, Spermidine, Formiminoglutamic acid, XA0004, Pantothenic acid, CMP, γ-Glu-Asp, Asn, Lactic acid, Uridine, Betaine, Tyr-Glu, Kynurenic acid, 2-Deoxyglucose 6-phosphate, Terephthalic acid, Cystine, 3′-CMP + 2′-CMP, N^ω^-Methylarginine, Guanine, Heptanoic acid, Isobutylamine, *o*-Coumaric acid + *p*-Coumaric acid, G3P, Kynurenine, UMP, 1,5-Gluconolactone, GMP, SAHC, 5-Hydroxylysine, Serotonin, 3′,5′-ADP, 2-PG, Spermine, Cysteine glutathione disulfide, GSSG, His-Glu, Aminoacetone, Octanoic acid, γ-Glu-Met, Oxoproline, ATP, SAM, γ-Glu-Gly, GTP, Fumaric acid, Guanosine, Inosine, Quinic acid, 3PSer, Isethionic acid, Adenosine, Hexanoic acid, Lys, 3-Hydroxy-2-methyl-4-pyrone, Ophthalmic acid, Glycerol, ADMA, S7P, AMP, UDP, Ser, dcSAM, *N*-Acetylgalactosamine + ManNAc + GlcNAc, 3-Mercaptolactic acid, 2-Hydroxyglutaric acid, 1-Methylhistidine + 3-Methylhistidine, XA0019, 11-Aminoundecanoic acid, *trans*-Glutaconic acid, Citraconic acid, *p*-Hydroxybenzoic acid, Acetylcholine, *N*-Acetylmethionine, Pelargonic acid, ADP, Theobromine, XC0089 + Nicotinamide riboside, Leu, 3-Hydroxy-3-methylglutaric acid, *N*-Acetylglucosylamine, Tyr, γ-Glu-His, Lauric acid, *N*6-Methyllysine, NAcGlcNP, Lauroylcarnitine, Malic acid, Arg, Decanoic acid, γ-Glu-Ile + γ-Glu-Leu, Glu, ADP-Glc + GDP-fucose, BTL, FAD, γ-Glu-Lys, CoA, PEP, γ-Glu-Gln, Glucuronic acid-3 + Galacturonic acid-3, GABA, 1H-Imidazole-4-propionic acid, His, Orotidine, γ-Glu, Phe, 3′-AMP, Uric acid, 3-Aminoisobutyric acid, DHAP, Pretonine, γ-Glu-Ser, *N*-Acetylalanine, XC0126, Succinyl AMP, Ascorbate 2-glucoside, Mucic acid, Thiaproline, Diethanolamine, and Agmatine	*O*-Acetylhomoserine + 2-Aminoadipic acid, ADP-Rib, Nicotinamide, Myoinositol 1-phosphate + Myoinositol 3-phosphate, Asp, Butyrylcarnitine, Putrescine, Thiamine, Hypoxanthine, Tyr-Arg_divalent, Acetylserine, Allantoic acid, Homoserine, IMP, UDP-GlcA, 4-GBA, Pipecolic acid, XC0040, Cadaverine, Methionine sulfoxide, Arg-Glu, F1,6P, MTA, Riboflavin, *N*-Acetylhistidine, Gly-Asp, 2-Methylserine, Nicotinic acid, 2,3-Diaminopropionic acid, cGMP, Ethanolamine, GDP, Octanoylcarnitine, XA0065, 2-Hydroxy-4-methylvaleric acid, G6P, F6P, 3-HBA, γ-Glu-Thr, Propionylcarnitine + XC0061, Trimethyllysine, dGuanosine, *N*-Acetylserine, Thr, Urocanic acid, XA0033, Ser-Glu, G1P, Sucrose 6′-phosphate, MIA, XC0016, Actinine, Pyridoxamine-P, β-Cyanoalanine, Phosphorylcholine, FMN, Citrulline, Cystathionine, Met, CDP-choline, Phe, Thymidine, 7-Methylxanthine, EAP, Saccharopine, Gln, and Adenine	1-Methyladenosine, *N*6-Acetyllysine, *N*-Methylproline, Octopamine + Dopamine, *N*1-Methylguanosine, *S*-Methylcysteine, *S*-Methylglutathione, Pterin, XC0132, DOPA, Xanthosine, Sebacic acid, Pro, 3-Methyladenine, γ-Glu-Cys, γ-Glu-Ala, *N*1-Acetylspermidine, Glucuronic acid-2 + Galacturonic acid-2, γ-Glu-Arg, Suberic acid, 2-OG, NAD+, 6-PG, SDMA, Cys, Homogentisic acid + *p*-Hydroxymandelic acid, Isovalerylcarnitine, Threonic acid, Succinic acid, Ru5P, *N*8-Acetylspermidine, 7-Methylguanine, PRPP, GSH, Hydroxyproline, Trp, *cis*-Hydroxyproline, Ile, Leu-Leu-Tyr, *N*-Formylkynurenine, GlcNAc-P, Biopterin, NicRN, Ala, Citric acid, Val, R5P, NADP+, γ-Glu-Val, ALCAR, Ornithine, 2-Aminoisobutyric acid + 2-Aminobutyric acid, Dyphylline, β-Ala, Choline, Gly, 4-Oxopyrrolidine-2-carboxylic acid, Azelaic acid, and Carnitine
Female	GDP, 6-PG, Stachydrine, BTL, XC0017, Sebacic acid, Isobutyric acid + Butyric acid, Glycerol, Azetidine 2-carboxylic acid, Ectoine, Myoinositol 2-phosphate, G1P, 11-Aminoundecanoic acid, β-Hydroxyisovaleric acid, Putrescine, FAD, Agmatine, Azelaic acid, Cystine, Deamido-NAD, Asp, γ-Glu-Citrulline, Dyphylline, R5P, UMP, MTA, 2-Hydroxyvaleric acid, dAdenosine + 5′-Deoxyadenosine, Hexanoic acid, CDP-choline, 4-GBA, Val, Pro-Pro, Cysteic acid, Biopterin, Isovaleric acid + *DL*-2-Methylbutyric acid + Valeric acid, GTP, Hypoxanthine, NADP+, XC0120, N-Acetylalanine, dcSAM, Tetrahydrouridine, Betaine, Diethanolamine, 3′,5′-ADP, 3-Methyladenine, Ru5P, Threonic acid, γ-Glu-Arg, NAD+, cGMP, UTP, NicRN, Xanthosine, Pimelic acid, ArgSuccinate, AMP, *N*6-Acetyllysine, Glycerol 2-phosphate, Pyruvic acid, Leu, Ophthalmic acid, *N*-Acetylserine, Galactosamine + Glucosamine, 3-Methoxytyrosine, Theobromine, Pyridoxal, Isobutylamine, Arg, 1-Aminocyclopropane-1-carboxylic acid + Homoserinelactone, ADP, SAHC, 7,8-Dihydrobiopterin, Ile, Asn, dTMP, Decanoic acid, 3-Ureidopropionic acid, Octanoylcarnitine, Choline, Gln, S-Methylmethionine, 3PSer, N-Acetyllysine, S7P, G3P, γ-Glu-Met, *cis*-Hydroxyproline, F1,6P, Leu-Leu-Tyr, cAMP, *N*-Acetylglucosylamine, 2-Methylserine, *N*-AcGlu, γ-Glu-Ornitine, Lauric acid, F6P, 3-Mercaptolactic acid, Terephthalic acid, Hexylamine, XA0017, Pterin, Glutaric acid + Methylsuccinic acid, *p*-Hydroxybenzoic acid, GPCho, 7-Methylguanine, Octanoic acid, XA0019, UDP, *m*-Hydroxybenzoic acid, Propionylcarnitine + XC0061, 5-Oxohexanoic acid, Pyridoxamine, 3′-CMP + 2′-CMP, *N*-Acetylaspartic acid, Pelargonic acid, Glucaric acid, *p*-Toluic acid + *m*-Toluic acid + *o*-Toluic acid, His-Glu, 3-Guanidinopropionic acid, Mevalolactone, and γ-Glu-Asp	Thiamine, MIA, Dimethylaminoethanol, Urocanic acid, *N*-Methylputrescine, Citrulline, 2-Deoxyglucose 6-phosphate, 5-Hydroxypentanoic acid, XC0065, Nicotinamide, Cadaverine, Myoinositol 1-phosphate + Myoinositol 3-phosphate, XA0033, 2-Hydroxy-4-methylvaleric acid, GSSG, Ethanolamine, TMP, Glyceric acid, Phosphorylcholine, Ornithine, GlcNAc-P, CTP, Penicillamine, CDP, 3-Methylguanine, 8-Hydroxyoctanoic acid, *o*-Hydroxybenzoic acid, 3-Hydroxy-3-methylglutaric acid, 3-PG, XC0132, CMP, NAcGlcNP, EAP, Inosine, ThPP, Riboflavin, Methionine sulfoxide, dCMP, XA0065, Nicotinic acid, Guanosine, ADP-Glc + GDP-fucose, PEP, Trehalose 6-phosphate, Met, GMP, Pyridoxamine-P, Gly-Asp, XC0040, FMN, IMP, 5-Hydroxylysine, Cysteine glutathione disulfide, Cystathionine, ATP, dGuanosine, Actinine, Uric acid, and Uridine	Ferulic acid, *o*-Coumaric acid + *p*-Coumaric acid, GSH, Allantoic acid, Adenine, 1-Methyladenosine, 2,3-Diaminopropionic acid, Shikimic acid, DOPA, Guanine, Homogentisic acid + *p*-Hydroxymandelic acid, Pipecolic acid, Mucic acid, Tyr, 2-Hydroxyglutaric acid, γ-Glu-Gly, SDMA, Indole-3-carboxylic acid, *S*-Methylcysteine, *O*-Acetylhomoserine + 2-Aminoadipic acid, Saccharopine, Succinyl AMP, γ-Glu-His, ADMA, Sulfotyrosine, Formiminoglutamic acid, γ-Glu-Asn, Isovalerylcarnitine, Citraconic acid, Trp, Glucuronic acid-1 + Galacturonic acid-1, *N*1-Methylguanosine, Ser-Glu, γ-Glu-Ser, XC0089 + Nicotinamide riboside, 3-Aminoisobutyric acid, Succinic acid, Adenosine, *trans*-Glutaconic acid, γ-Glu-Thr, *trans*-Cinnamic acid, Quinic acid, Isethionic acid, DHMA, γ-Glu-Phe, *N*-Formylkynurenine, *O*-Acetylserine, Phe, Lys, XC0126, Gly-Gly, *S*-Methylglutathione, Ascorbate 2-glucoside, Malic acid, *S*-Carboxymethylcysteine, Fumaric acid, XC0016, Carnitine, XC0071, ALCAR, Pyridoxine, N^ω^-Methylarginine, *N*1-Acetylspermidine, γ-Glu-Val, Pro, Aminoacetone, *N*6-Methyllysine, *N*5-Ethylglutamine, *N*8-Acetylspermidine, Oxoproline, Thiaproline, γ-Glu-Gln, Triethanolamine, γ-Glu-Ile + γ-Glu-Leu, Arg-Glu, γ-Glu-Lys, Carboxymethyllysine, γ-Glu-Ala, *N*-Methylproline, Mevalonic acid, 3′-AMP, Kynurenine, 3-HBA, UDP-GlcA, Homoserine, Imidazole-4-acetic acid, His, Spermidine, Hydroxyproline, β-Ala, Isocitric acid, β-Cyanoalanine, Heptanoic acid, GABA, 2-OG, Metronidazole Glucuronic acid-3 + Galacturonic acid-3, G6P, Trimethyllysine, Citric acid, *cis*-Aconitic acid, Glucuronic acid-2 + Galacturonic acid-2, BAPN, *N*-Acetylgalactosamine + ManNAc + GlcNAc, 7-Methylxanthine, Lactic acid, 2-Aminoisobutyric acid + 2-Aminobutyric acid, Spermine, 2,3-Dihydroxybenzoic acid, Ala, Dihydrouracil, Homocitrulline, Phosphoramidon, 1-Methylhistidine + 3-Methylhistidine, Val, Gly, Gluconic acid, Thr, XA0004, 1,5-Gluconolactone, Thymidine, SAM, Uracil, Pantothenic acid, Ser, 4-Oxopyrrolidine-2-carboxylic acid, Glu, and 3-Hydroxy-2-methyl-4-pyrone

This table is generated based on *p* values computed by Welch’s test using relative peak area.

**Table 3:** Sample of *H. schachtii* and *H. trifolii* used for metabolite analysis.

Sample Name	Replicates	Dried Extract (mg)	Solvent (ACN:H_2_O;1:1) (µL)
*H. schachtii* Egg	HS-egg-1	24.6	1200
	HS-egg-2	27.9	1200
	HS-egg-3	27.4	1200
*H. schachtii* Juvenile-2	HS-J2-1	20.8	900
	HS-J2-2	28.3	1200
	HS-J2-3	24.9	1200
*H. schachtii* Female	HS-F-1	25.1	1200
	HS-F-2	19.1	900
	HS-F-3	27.5	1200
*H. trifolii* Egg	HT-egg-1	20.7	900
	HT-egg-2	26.1	1200
	HT-egg-3	25.3	1200
*H. trifolii* Juvenile-2	HT-J2-1	22.5	900
	HT-J2-2	29.7	1200
	HT-J2-3	30.1	1500
*H. trifolii* Female	HT-F-1	24.7	1200
	HT-F-2	14.4	600
	HT-F-3	12.3	600

HS, Heterodera schachtii; HT, Heterodera trifolii; J2, Juvenile 2; F, Female.

## Data Availability

Data supporting the results are included in this published article and its Supplementary Materials.

## Supplementary Materials

The following are available online at https://www.mdpi.com/article/10.3390/ijms221910488/s1.
