# Supplementary material for: Metabolic Analysis of the Development of the Plant-Parasitic Cyst Nematodes Heterodera schachtii and Heterodera trifolii by Capillary Electrophoresis Time-of-Flight Mass Spectrometry
Source: Int J Mol Sci. 2021 Sep 28;22(19):10488. doi: 10.3390/ijms221910488 (PMC8508704; doi:10.3390/ijms221910488)
Supplement: Supplementary file 1 [file ijms-22-10488-s001.zip › Supplementary Figures S1, S2, and S3.pdf]

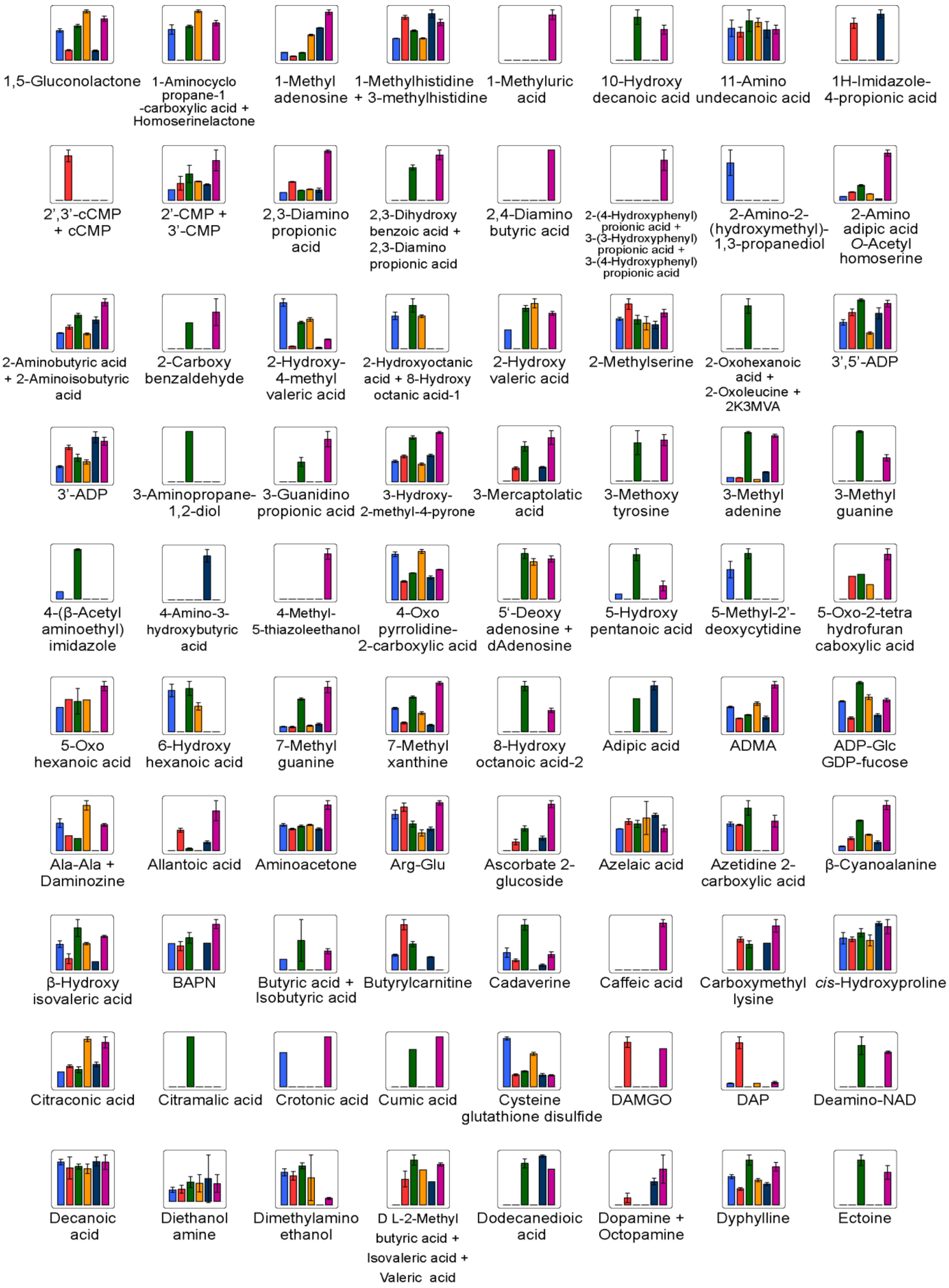

Figure S1a. Metabolites not shown on the pathway map. The bars/lines represent peak areas of each metabolite in *H. schachtii* egg (blue), *H. schachtii* J2 (red), *H. schachtii* female (green), *H. trifolii* egg (orange), *H. trifolii* J2 (mazarine), and *H. trifolii* female (purple), respectively.

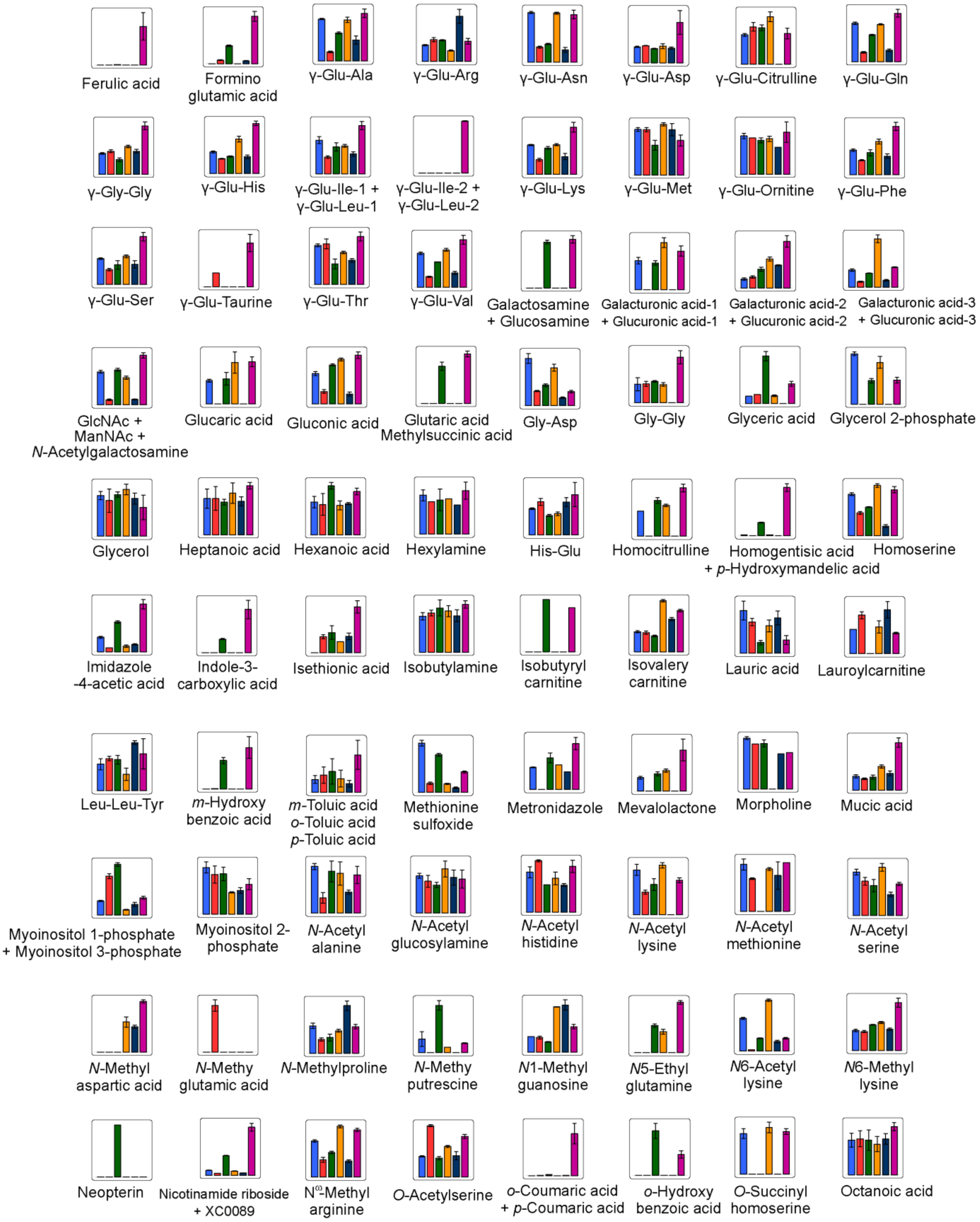

Figure S1b. Metabolites not shown on the pathway map. The bars/lines represent peak areas of each metabolite in *H. schachtii* egg (blue), *H. schachtii* J2 (red), *H. schachtii* female (green), *H. trifolii* egg (orange), *H. trifolii* J2 (mazarine), and *H. trifolii* female (purple), respectively.

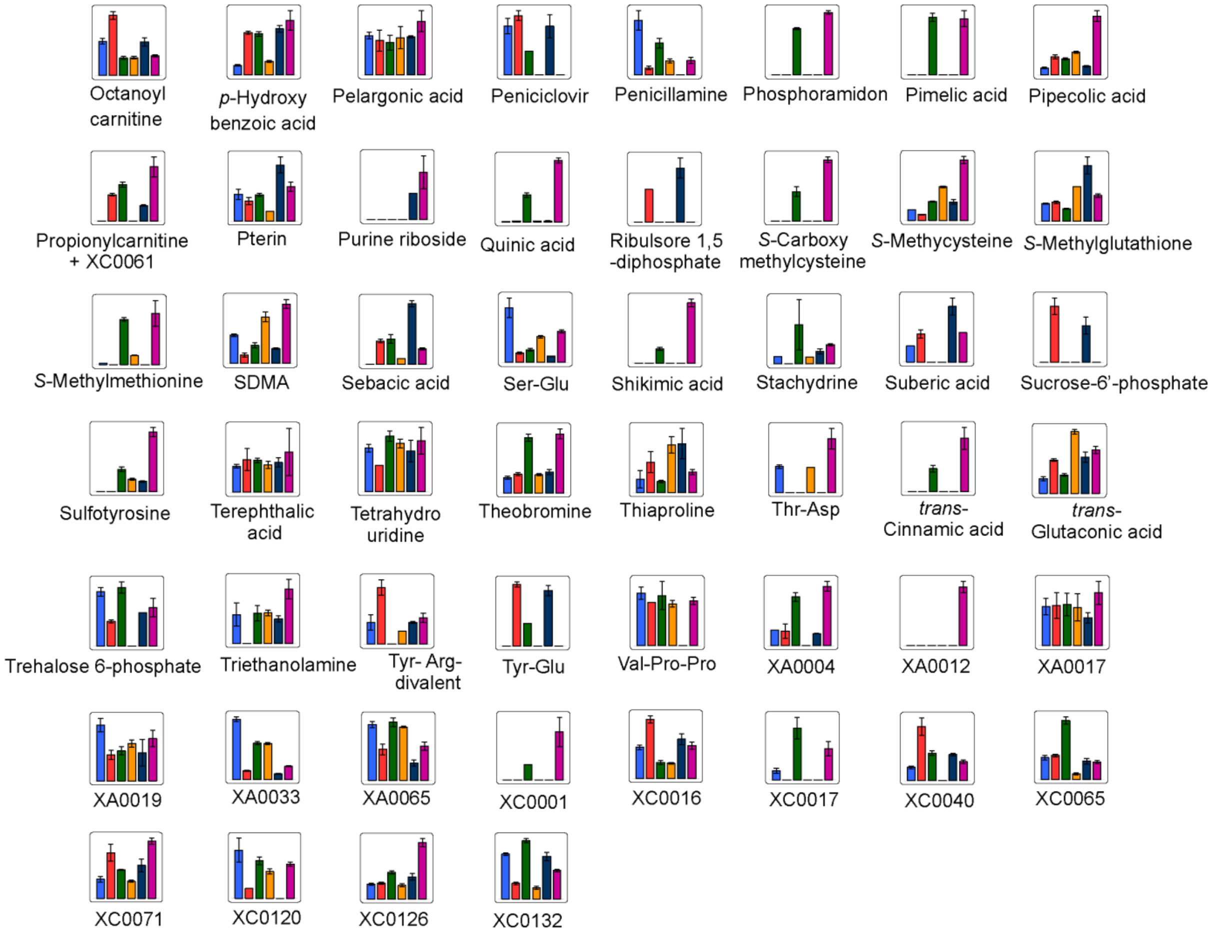

Figure S1c. Metabolites not shown on the pathway map. The bars/lines represent peak areas of each metabolite in *H. schachtii* egg (blue), *H. schachtii* J2 (red), *H. schachtii* female (green), *H. trifolii* egg (orange), *H. trifolii* J2 (mazarine), and *H. trifolii* female (purple), respectively.

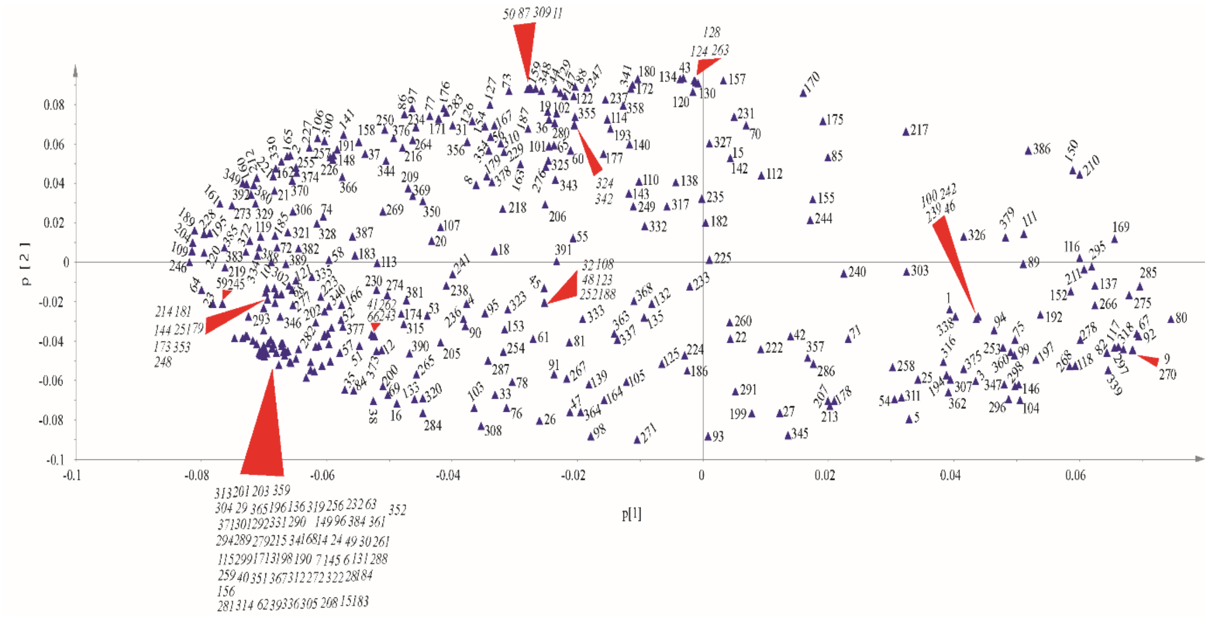

Figure S2. PCA loading scatter plot of peaks detected in the egg, J2, and female stages of *H. schachtii* and *H. trifolii*

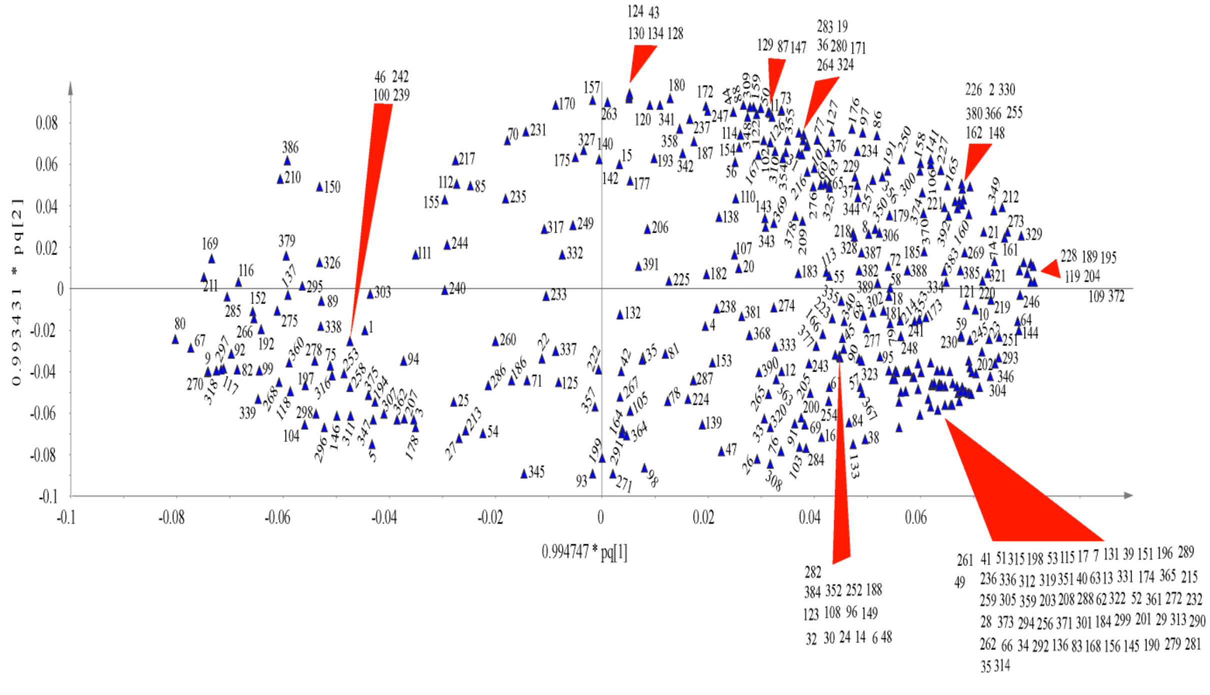

Figure S3. OPLS-DA loading scatter plot of peaks detected in the egg, J2 and female stages of *H. schachtii* and *H. trifolii*
